# Production of a selective antibacterial fatty acid against *Staphylococcus aureus* by *Bifidobacterium* strains

**DOI:** 10.20517/mrr.2022.24

**Published:** 2023-02-22

**Authors:** Hiroshi Kikukawa, Toshihiro Nagao, Mitsuki Ota, Shigeo Takashima, Kohji Kitaguchi, Emiko Yanase, Sadatoshi Maeda, Kiyotaka Y. Hara

**Affiliations:** ^1^Graduate Division of Nutritional and Environmental Sciences, University of Shizuoka, Shizuoka 422-8526, Japan.; ^2^School of Food and Nutritional Sciences, University of Shizuoka, Shizuoka 422-8526, Japan.; ^3^Research Division of Biomaterials and Commodity Chemicals, Osaka Research Institute of Industrial Science and Technology, Osaka 536-8553, Japan.; ^4^Graduate School of Natural Science and Technology, Gifu University, Gifu 501-1193, Japan.; ^5^Institute for Glyco-core Research (iGCORE), Gifu University, Gifu 501-1193, Japan.; ^6^Faculty of Applied Biological Sciences, Gifu University, Gifu 501-1193, Japan.; ^7^The United Graduate School of Veterinary Sciences, Gifu University, Gifu 501-1193, Japan.

**Keywords:** *Bifidobacterium*, screening, hexadecenoic acid, selective antibacterial activity, *Staphylococcus aureus*, *Staphylococcus epidermidis*

## Abstract

**Aims:** C16 monounsaturated fatty acid (C16:1) show antibacterial activity against *Staphylococcus aureus*, a pathogen associated with various diseases such as atopic dermatitis and bacteremia, while the compound does not exhibit antibacterial activity against *Staphylococcus epidermidis*, an epidermal commensal that inhibits the growth of *S. aureus*. In this study, we aimed to find bifidobacterial strains with the ability to produce C16:1 and to find a practical manner to utilize C16:1-producing strains in industry.

**Methods:** Various *Bifidobacterium* strains were screened for their content of C16:1. The chemical identity of C16:1 produced by a selected strain was analyzed by gas chromatography-mass spectrometry (GC-MS) and liquid chromatography-mass spectrometry (LC-MS). Medium components that affect the C16:1 content of the selected strain were investigated. Antibacterial activity against staphylococci was compared between the authentic C16:1 isomers and total fatty acids (TFA) extracted from the selected strain.

**Results:** *B. adolescentis* 12451, *B. adolescentis* 12-111, *B. boum* JCM 1211, and *Bifidobacterium* sp. JCM 7042 showed high C16:1 content among the tested strains. TFA extracted from *Bifidobacterium* sp. JCM 7042 contained C16:1 at 2.3% as the fatty acid constituent (2.4 mg/L of broth). Through GC-MS and LC-MS analyses, the C16:1 synthesized by *Bifidobacterium* sp. JCM 7042 was identified as 7-*cis*-hexadecenoic acid (7-*cis*-C16:1). The authentic 7-*cis*-C16:1 showed strong and selective antibacterial activity against *S. aureus*, similar to 6-*cis*-C16:1, with a minimum inhibitory concentration (MIC) of < 10 µg/mL. Components that increase C16:1 productivity were not found in the MRS and TOS media; however, Tween 80 was shown to considerably reduce the C16:1 ratio in TFA. Antibacterial activity against *S. aureus* was observed when the TFA extracted from *Bifidobacterium* sp. JCM 7042 contained high level of 7-*cis*-C16:1 (6.1% in TFA) but not when it contained low level of 7-*cis*-C16:1 (0.1% in TFA).

**Conclusion:** The fatty acid, 7-*cis*-C16:1, which can selectively inhibit the *S. aureus* growth, is accumulated in TFA of several bifidobacteria. The TFA extracted from cultured cells of *Bifidobacterium* sp. JCM 7042 demonstrated antibacterial activity. From a practical viewpoint, our findings are important for developing an efficient method to produce novel skin care cosmetics, functional dairy foods, and other commodities.

## INTRODUCTION

Bifidobacteria are Gram-positive, nonspore-forming, nonmotile, and catalase-negative anaerobic bacteria belonging to the phylum Actinobacteria^[1]^. The bacteria ferment sugars primarily to lactic acid and acetic acid *via* a unique metabolic pathway, the so-called bifido shunt^[2]^. There is accumulating evidence showing that bifidobacteria contribute to maintaining human health^[3]^. The probiotic traits exerted by bifidobacteria are utilized in functional food production, where fermented milk is one of the most popular foods to be supplemented. So far, most of the studies on bifidobacteria have been conducted to understand their diversified saccharide metabolism, which is highlighted by plant poly/oligosaccharides and human milk oligosaccharide assimilation pathways^[4-6]^. Bacteriocin production has also been reported for some bifidobacterial strains^[7]^. On the other hand, studies on lipid metabolism in bifidobacteria are limited^[8,9]^ and the pathway for fatty acid biosynthesis in bifidobacteria remains poorly understood^[8,10-13]^.

Staphylococci constitute a group of major commensal/pathogenic microorganisms of the human skin. Among them,* Staphylococcus aureus* (*S. aureus*) is a well-known coagulase-positive pathogen associated with many diseases, *e.g.,* atopic dermatitis, bacteremia, and food poisoning^[14-18]^. *S. aureus* is rarely detected on healthy skin (an average of 1.1% of all skin bacteria), but becomes predominant (average: 65%) on the skin of patients with atopic dermatitis^[19]^. It produces several toxins and proteases, which aggravate skin inflammation by disrupting the epidermal barrier^[15,20]^. In contrast, *Staphylococcus epidermidis* (*S. epidermidis*) is a coagulase-negative commensal that is detected on the skin of both healthy individuals and patients with atopic dermatitis. *S. epidermidis* has positive effects on skin health through the production of a protein that stimulates the secretion of antimicrobial peptide β-defensin from keratinocytes, a phenol-soluble modulin that inhibits the growth of *S. aureus*, a serine protease that inhibits biofilm formation by *S. aureus*, and lipoteichoic acid that represses inflammatory cytokine release from keratinocytes^[21-27]^. Several studies thus indicate that eradication of* S. aureus* from skin without affecting colonization by coagulase-negative *Staphylococcus* species is one of the promising strategies for treatment of atopic dermatitis^[17,18]^. In this regard, isolation of compounds with high selectivity towards harmful *S*.* aureus* warrants further research.

Recent studies have revealed that C16 monounsaturated fatty acids (hexadecenoic acid, C16:1) such as 6-*cis*-C16:1 (sapienic acid) and 9-*cis*-C16:1 (palmitoleic acid) possess a selective antibacterial activity against *S. aureus*^[28-31]^, which has made C16:1 an attractive target of pharmaceutical therapy. Sapienic acid, which is present in human sebum, is considered to control the skin microbiota as a natural antimicrobial agent. Indeed, the level of sapienic acid was lower in atopic dermatitis patients than in healthy individuals and was inversely correlated to the abundance of *S. aureus*^[32,33]^. This compound is, however, not easily obtained in nature because it is scarcely found in natural oils. Palmitoleic acid is a known component of several natural oil such as macadamia nut oil and sea buckthorn fruit oil, but the amount is too low to meet industrial use, either.

Given this, in this study, we first screened bifidobacterial strains for their ability to produce C16:1. Then, the antibacterial activity towards *Staphylococcus* strains was examined by using total fatty acids (TFA) extracted from the selected strain. The results revealed the possibility of the strain for practical use to develop skin care cosmetics and functional foods.

## METHODS

### Strains and culture conditions

Bacterial strains were purchased from the Biological Resource Center, National Institute of Technology and Evaluation (NBRC) (Tokyo, Japan), and the Japan Collection of Microorganisms in Riken BioResource Research Center (Ibaraki, Japan). Bifidobacterial type-strain cultures (JCM 1190, 1192, 1194, 1195, 1200, 1207, 1211, 1222, 1255, 1275, 1302, 6291, 7042, 8222, 10602, 12451, and 31944, and NCC 2705),* B. adolescentis* labo-stock cultures (12451, 3-117, 12-114, 12-111, 4-2, 4-16, 4-58, and 9-124), and staphylococci (*S. aureus* NBRC 100910^T^, 12732, 13276, and 14462, and *S. epidermidis* NBRC 100911^T^ and 12993, and ATCC 49134) were used in this study.

*Bifidobacterium* strains were cultured in 12 mL of MRS medium for two days at 37 °C and used for screening for C16:1 content. TOS medium consisting of 10 g/L galactooligosaccharide, 10 g/L Tryptone, 1.0 g/L Yeast extract, 3.0 g/L K_2_HPO_4_, 4.8 g/L KH_2_PO_4_, 3.0 g/L (NH_4_)_2_SO_4_, and 0.2 g/L MgSO_4_·7H_2_O supplemented with 0.5 g/L L-cysteine was also used for cultivation. NB medium was used for the cultivation of staphylococci and for dilution of the staphylococci cultures in an antibacterial assay which contained 0.5% extra bonito, 1.0% hi-polypeptone, and 0.5% NaCl (pH: 6.0).

Lactobacilli MRS broth, Tryptone, Yeast extract, and galactooligosaccharide were purchased from Difco, Becton, Dickinson and Co. (Sparks, MD, USA). Other reagents and solvents were purchased from FUJIFILM Wako Pure Chemical Corporation (Osaka, Japan).

### Fatty acid analysis

Fatty acids extracted in the form of methyl ester from bifidobacteria were prepared and analyzed using a previously reported method with minor modifications^[34]^. TFA is represented by the extracted fatty acid methyl ester. Following cultivation, bacterial cells were harvested by centrifugation (3260 × *g*) at 4 °C for 20 min, washed twice with 0.85% NaClaq, and dried at 100 °C for 2.5 h. After measuring the dried cell weight, the dried cells were directly transmethylated with 10% methanolic HCl at 55 °C for 2.5 h. Tricosanoic acid (C23:0) was used as an internal standard. The resultant fatty acid methyl esters were extracted with *n*-hexane before being concentrated and then analyzed by gas chromatography (GC) equipped with a TC-70 capillary column (GL Sciences Inc., Tokyo, Japan) and a flame ionization detector. The initial column temperature of 160 °C was raised to 230 °C at 2 °C/min and maintained for 10 min. The injector and detector were operated at 250 °C.

### Identification of the chemical structure

The TFA from *Bifidobacterium *sp. JCM 7042 were derivatized into 4,4-Dimethyloxazoline (DMOX) and subsequently analyzed by gas chromatography-mass spectrometry (GC-MS) as described previously^[35,36]^. The position of the double bond of C16:1 was analyzed using the plasma-mediated modification method by liquid chromatography-mass spectrometry (LC-MS) as previously reported^[37]^. For LC-MS analysis, fatty acid samples were analyzed in the form of free fatty acid (FFA).

### Preparation of FFA samples extracted from *Bifidobacterium* sp. JCM 7042

To obtain FFAs for the LC-MS analysis and antibacterial assay, bacterial cells were harvested by centrifugation (3260 × *g*) at 4 °C for 20 min, washed twice with 0.85% NaClaq, and directly acid-hydrolyzed. Briefly, a pellet of *Bifidobacterium* cells was dissolved in 400 µL of acetonitrile and 50 μL of 5.0 N HCl in a glass tube. The sample was lysed by vortex and incubated at 100 °C for one hour. After cooling to room temperature, 800 μL of *t*-butyl methyl ether, 100 μL of methanol, and 400 μL of H_2_O were added, and the mixture was vortexed for 1 min. After centrifugation at 3260 × *g* for 5 min, the upper organic phase was collected. Subsequently, 800 μL of water was added, and the sample was vortexed again for 1 min. After phase separation at 3260 × *g* for 5 min, the upper organic phase containing FFAs was collected. The collected organic phase was evaporated under a stream of nitrogen gas, and the recovered FFAs were reconstituted in 100 μL of acetone before being subjected to LC-MS analysis.

### Antibacterial assay

The antibacterial activity of FFAs was evaluated by determining minimum inhibitory concentration (MIC) as described in a previous study^[38]^. *S. aureus* and* S. epidermidis* cells collected at the exponential growth phase were diluted to a concentration of 2.0 × 10^4^ CFU/mL with the NB medium. The cell suspensions were dispensed into 96-well titer plate, to which fatty acids dissolved in dimethyl sulfoxide or ethanol (10 mg/ml) were added. The fatty acid concentrations were varied between 1.0 and 2000 μg/mL. The assays were carried out in biological triplicate. The lowest concentration (highest dilution) required to prevent the growth of microorganisms was regarded as the MIC.

## RESULTS

### Screening of *Bifidobacterium* strains for C16:1 content

As a preliminary screening, approximately one hundred microbial strains collected from soil samples and laboratory stocks, including Enterobacteriaceae, Saccharomycetaceae, Bifidobacteriaceae, Nocardiaceae, and some filamentous fungi, were screened for their content of C16:1; however, no characteristic C16:1 production was observed for any of the strains, except for the ones belonging to the genes *Bifidobacterium* (data not shown). Therefore, as the first screening, we examined the fatty acid content of 25 *Bifidobacterium* strains by GC [Table 1]. MRS medium was used for cultivation. As a result, several strains of *B. adolescentis*, *B. boum* JCM 1211, and *Bifidobacterium *sp. JCM 7042 strains were found to show high unknown C16:1 content that is not 9-*cis*-C16:1. Among them, *B. boum* JCM 1211 had the highest C16:1 ratio (2.7%) in TFA, while *Bifidobacterium *sp. JCM 7042 showed the highest amounts of TFA and C16:1 (106.6 and 2.4 mg/L of broth, respectively). Other species, such as *B. bifidum, B. animalis, B. indicum, *and* B. longum*, did not contain the C16:1 as a fatty acid constituent.

**Table 1:** The C16:1^*a*^ content of *Bifidobacterium* strains (1^st^ screening)

**Strain**	**TFA^*b*^ (mg/L)**	**C16:1 (%)**	**C16:1 (mg/L)**	**Strain**	**TFA^*b*^ (mg/L)**	**C16:1 (%)**	**C16:1 (mg/L)**
*B. bifidum *JCM 1255	24.7	0	0	*B. breve *JCM 1192	67.8	0.5	0.3
*B. adolescentis* JCM 1275	43.4	0	0	*B. catenulatum* subsp.* catenulatum *JCM 1194	36.6	0	0
*B. adolescentis* 12451	55.8	1.8	1.0	*B. dentium* JCM 1195	44.4	0.6	0.3
*B. adolescentis* 3-117	54.4	0.5	0.3	*B. gallunarum* subsp.* gallunarum* JCM 6291	20.2	0.7	0.1
*B. adolescentis* 12-111	106.0	2.3	2.4	*B. indicum* JCM 1302	23.4	0	0
*B. adolescentis* 12-114	39.4	0.9	0.4	*B. longum *subsp. *longum *JCM 31944	65.4	0	0
*B. adolescentis* 4-2	57.4	0	0	*B. longum* subsp.* infantis* JCM 1222	56.0	0	0
*B. adolescentis* 4-16	19.6	0	0	*B. longum *NCC 2705	93.5	0	0
*B. adolescentis* 4-58	39.0	0.6	0.2	*B. pseudocatenulatum* JCM 1200	40.9	0	0
*B. adolescentis* 9-124	33.4	0.3	0.1	*B. ruminantium* JCM 8222	74.9	0.7	0.5
*B. animalis* subsp. *animalis* JCM 1190	44.7	0.0	0	*B. thermophilum* JCM 1207	68.1	0.8	0.5
*B. animalis* subsp.* lactis *JCM 10602	35.2	0.6	0.2	*Bifidobacterium* sp. JCM 7042	106.6	2.3	2.4
*B. boum *JCM 1211	77.5	2.7	2.1				

a C16:1: unknown C16:1, not including 9-*cis*-16:1. C16:1: C16 monounsaturated fatty acid; ^*b*^TFA: total fatty acids. The values obtained from a single technical replicate are shown.

**Figure 1:**
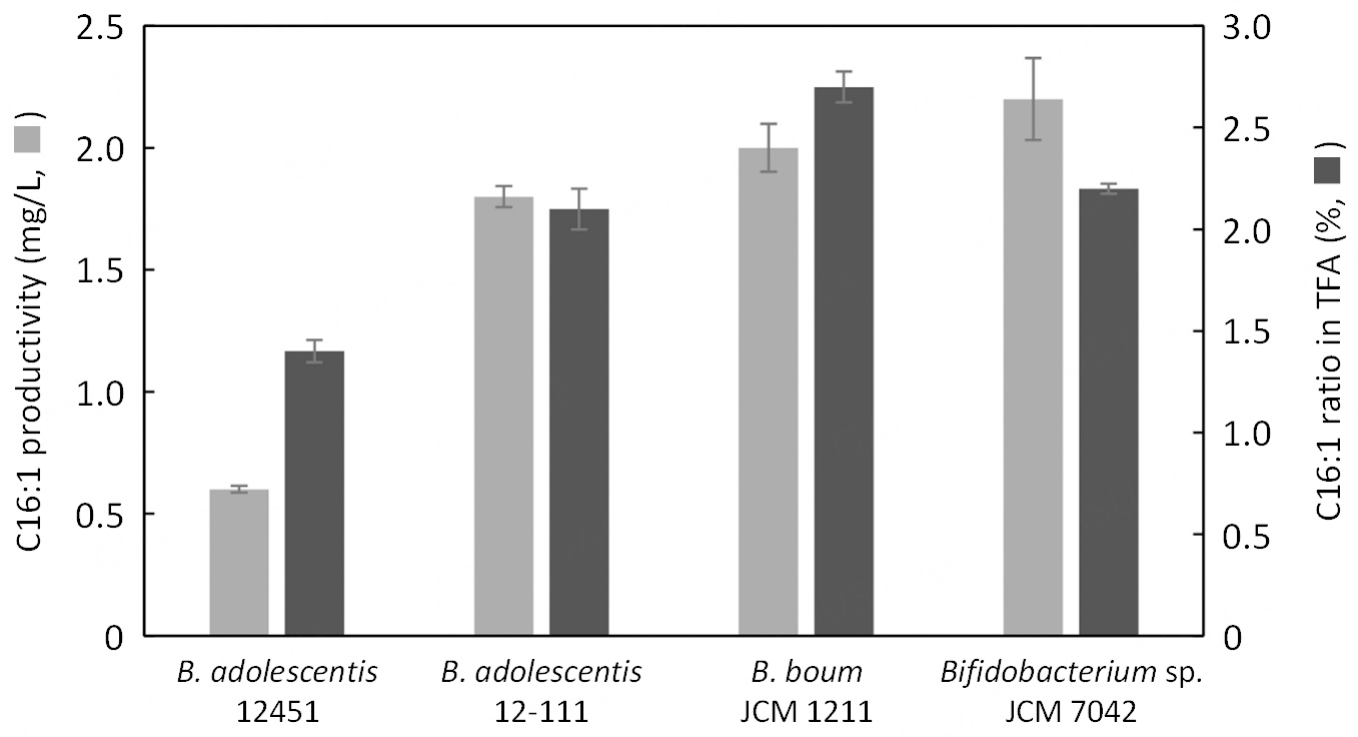
The C16:1 content of the selected *Bifidobacterium* strains (2^nd^ screening). The gray bar indicates C16:1 content (mg/L of broth), while the black bar indicates the C16:1 percentage in total fatty acids (TFA). Data are mean ± SD of three independent assays, represented by the bars and whiskers. C16:1: unknown C16:1, not including 9-*cis*-16:1. C16:1: C16 monounsaturated fatty acid.

*B. adolescentis* 12451, *B. adolescentis* 12-111, *B. boum* JCM 1211, and *Bifidobacterium* sp. JCM 7042 were then used for the second screening, in which TOS broth was used for cultivation [Figure 1]. *B. boum* JCM 1211 and *Bifidobacterium* sp. JCM 7042 showed comparable C16:1 content with *Bifidobacterium* sp. JCM 7042 being the highest. Based on the results obtained from the first and second screenings, we selected *Bifidobacterium* sp. JCM 7042 for further study.

### Chemical identity of the C16:1 extracted from *Bifidobacterium* sp. JCM 7042 cells

**Figure 2:**
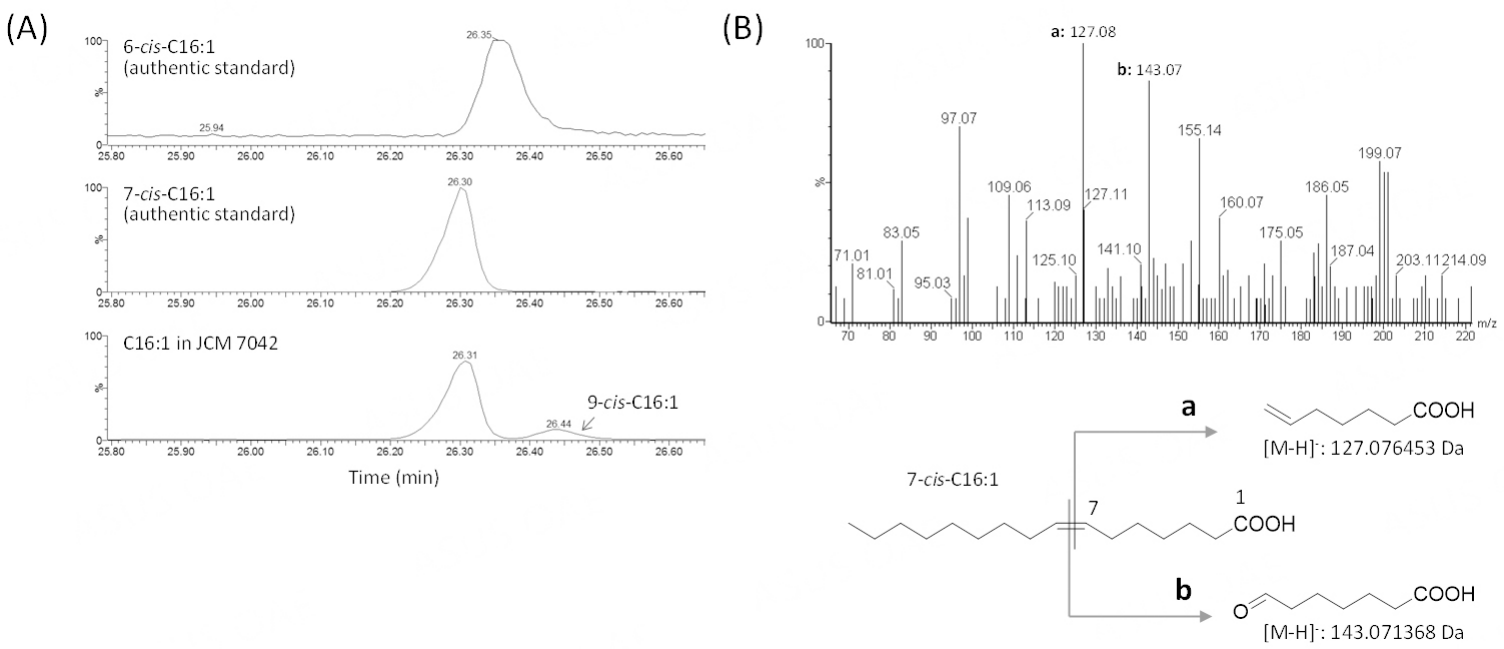
Chemical identity of C16:1 produced by *Bifidobacterium *sp. JCM 7042. (A) GC-MS chromatograms of DMOX-derivatized authentic standards (upper two panels) and TFA obtained from the JCM 7042 strain (bottom panel); (B) the spectrum of C16:1 obtained from the JCM 7042 strain is shown with the CID fragmentation pattern of 7-*cis*-hexadecenoic acid. The peaks of the fragments generated are indicated by “a” and “b”. CID: Collision-induced dissociation.

To examine the chemical identity of unknown C16:1, the DMOX derivatives of TFA extracted from the JCM 7042 strain were analyzed by GC-MS [Figure 2A]. The analysis revealed that the molecular mass of the C16:1 was the same as that of C16:1 authentic standards (*m/z* 307). The elution time of the C16:1 corresponded to that of the 7-*cis*-C16:1 authentic standard but not to that of 6-*cis*-C16:1 or 9-*cis*-C16:1.

To determine the position of the double bond in the C16:1 obtained from the JCM 7042 strain, FFAs prepared from TFA were analyzed by LC-MS. For that analysis, FFAs were not derivatized. *Via* the plasma-mediated modification of the carbon-carbon double bond into epoxide, followed by collision-induced dissociation (CID) at the epoxide position, the unsaturated fatty acid was intramolecularly dissociated into several fragments. The positions of double bonds in the unsaturated fatty acid were then determined from the structure of the generated fragments. C16:1 extracted from the JCM 7042 strain was dissociated into 6-heptenoic acid ([M-H]^-^: *m/z *127.08) and 7-oxoheptanoic acid ([M-H]^-^: *m/z* 143.07) [Figure 2B]. The CID fragmentation pattern revealed that the C16:1 has a double bond at the Δ7 position. Accordingly, the C16:1 from the JCM 7042 strain was identified as 7-*cis*-hexadecenoic acid.

### Antibacterial activities of authentic C16:1 standards against staphylococci

**Figure 3:**
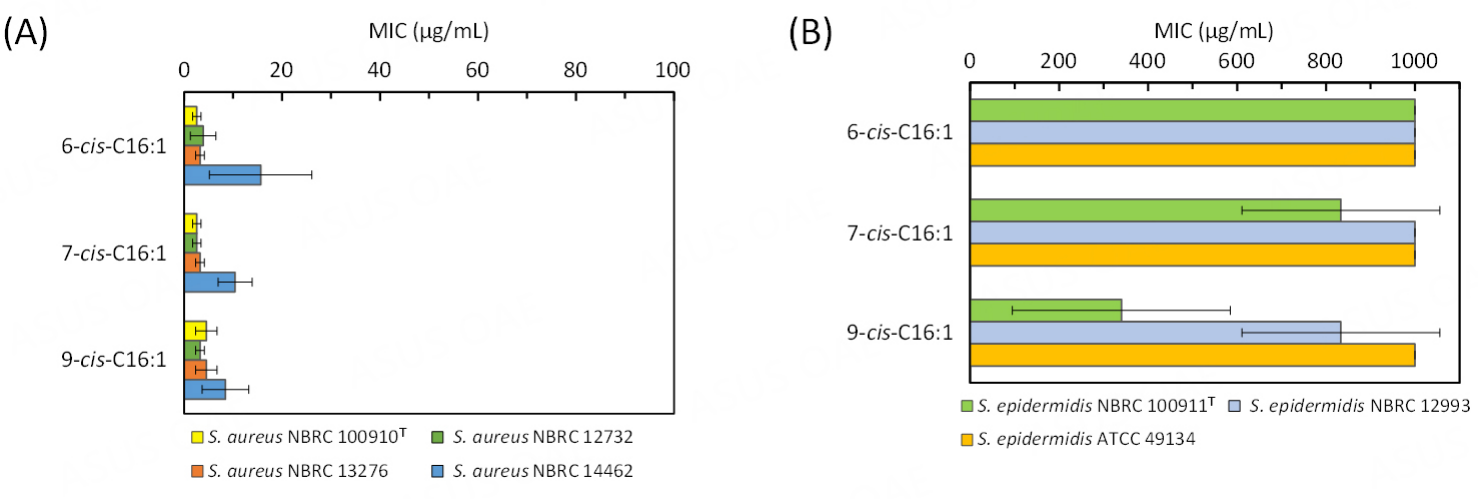
Antibacterial activities of C16:1 authentic standards against *S. aureus* (A) and *S. epidermidis* (B) strains. MIC values of 6-*cis*-C16:1, 7-*cis*-C16:1, and 9-*cis*-C16:1 isomers for *S. aureus* and *S. epidermidis* were determined. Data are mean ± SD of three independent assays, represented by the bars and whiskers. MIC: Minimum inhibitory concentration.

Selective antibacterial activity against *S. aureus* has been shown for 6-*cis*-C16:1 (sapienic acid) and 9-*cis*-C16:1^[28-31]^. To examine whether 7-*cis*-C16:1 shows similar activity, the antibacterial assay was carried out using the three authentic C16:1 standards [Figure 3A and B]. Four *S. aureus* strains (NBRC 100910^T^, NBRC 12732, NBRC 13276, and NBRC 14462) and three *S. epidermidis* strains (NBRC 100911^T^, NBRC 12993, and ATCC 49134) were used for the assay.

Each authentic standard of C16:1 showed strong antibacterial activity against the three *S. aureus* strains (NBRC 100910^T^, NBRC 12732, and NBRC 13276) at a MIC of < 5.0 µg/mL [Figure 3A]. *S. aureus* NBRC 14462 showed slightly higher tolerance to all C16:1 isomers with the MIC values around 10 µg/mL. On the other hand, 6-*cis*-C16:1 showed no antibacterial activity against the three tested *S. epidermidis* strains with MIC values of > 1000 µg/mL [Figure 3B]. 7-*cis*-C16:1 also did not show high antibacterial activity against the three *S. epidermidis* strains, although the growth inhibition for the NBRC 100911^T^ strain was observed at the concentration of > 800 µg/mL. 9-*cis*-C16:1 showed moderate antibacterial activity against *S*. *epidermidis* NBRC 100911^T^ at a MIC of 340 µg/mL. The growth inhibition for *S*. *epidermidis* NBRC 12993 was observed at the concentration of > 800 µg/mL. The results indicated that 6-*cis*-C16:1 and 7-*cis*-C16:1 possess higher selectivity towards *S. aureus* than 9-*cis*-C16:1 and that the antibacterial activity of the former two isomers against *S. aureus* is over 100-fold higher than that for *S*. *epidermidis*.

### Medium components that affect C16:1 content of the JCM 7042 strain

**Figure 4:**
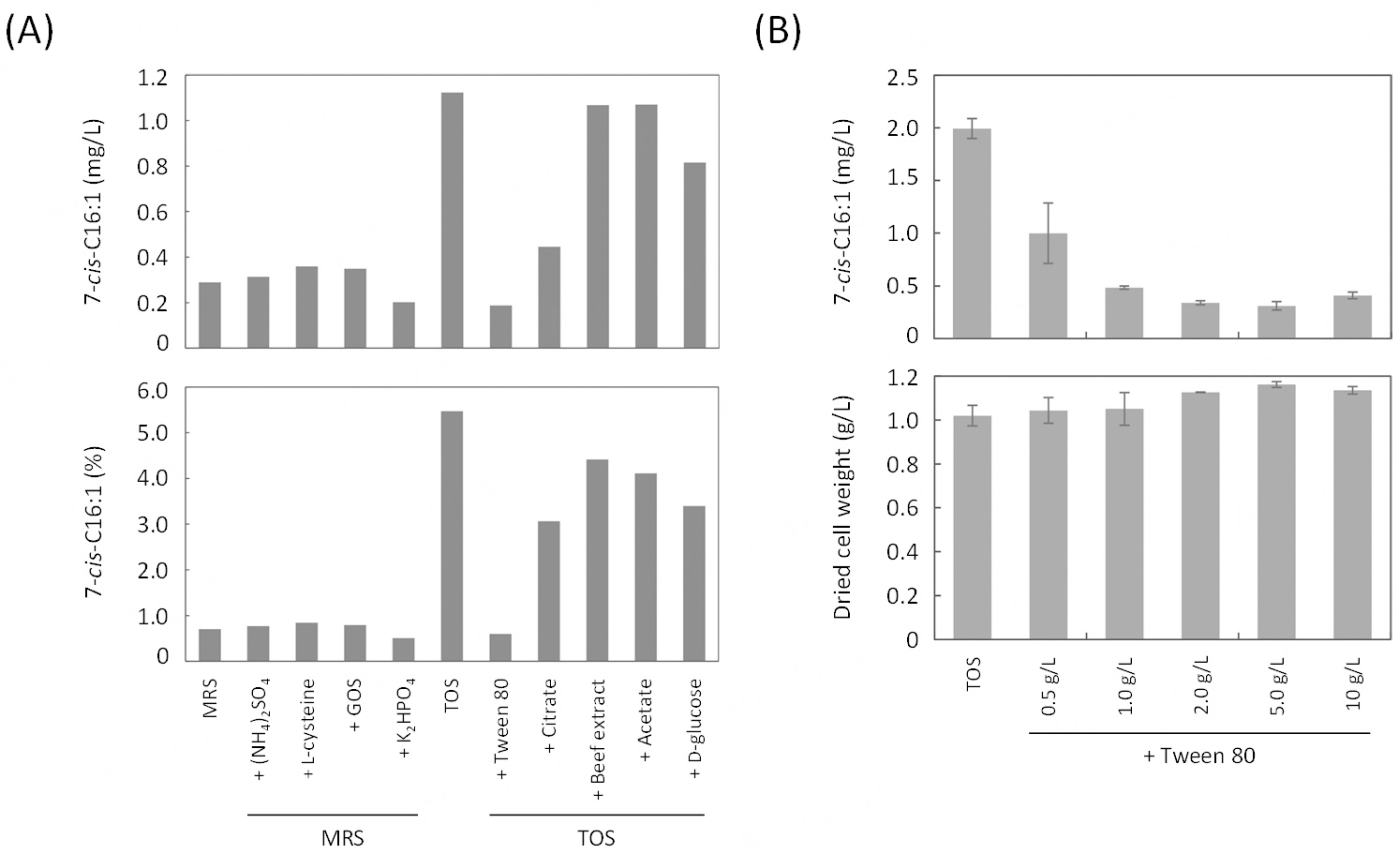
Effect of medium components on the 7-*cis*-C16:1 content in the JCM 7042 cells. (A) Each of TOS and MRS medium components was added to the MRS and TOS media, respectively, at the concentration of 3.0 g/L for (NH_4_)_2_SO_4_; 0.5 g/L for L-cysteine; 10 g/L for galactooligosaccharide (GOS); 3.0 g/L for K_2_HPO_4_, 1.0 g/L for Tween 80, 2.0 g/L for ammonium citrate (Citrate); 10 g/L for beef extract; 5.0 g/L for sodium acetate (Acetate); and 20 g/L for glucose; (B) effect of Tween 80 on the 7-*cis*-C16:1 content and post-culture cell biomass of the JCM 7042 strain.

Medium components may affect microbial metabolism. We used TOS and MRS media for cultivation of the JCM 7042 strain and compared the 7-*cis*-C16:1 content in the cells. The results clearly showed that the strain produced more 7-*cis*-C16:1 both in the amount and in the ratio in TFA when grown in TOS medium than grown in MRS medium [Figure 4A]. Then, we supplemented the MRS medium with each of TOS medium components or supplemented the TOS medium with each of MRS medium components to examine how each of the components affects the 7-*cis*-C16:1 content in the cells [Figure 4A]. The results showed that neither of the TOS components, (NH_4_)_2_SO_4_, L-cysteine, galactooligosaccharide, nor K_2_HPO_4 _did increase 7-*cis*-C16:1 content of the cells when MRS-based medium was used. However, the addition of the MRS components, Tween 80, citrate, and glucose to the TOS medium markedly decreased 7-*cis*-C16:1 content both in the amount and in the ratio in TFA. Especially, the addition of Tween 80, a surfactant, drastically reduced the content by 10-fold (1.1 mg/L and 5.5% in TFA to 0.2 mg/L and 0.6% in TFA) to a level similar to that obtained when grown in MRS. The effect of Tween 80 on the 7-*cis*-C16:1 productivity was dose-dependent, but its addition to the TOS medium did not affect post-culture cell biomass [Figure 4B].

### Antibacterial ability of TFA prepared from the JCM 7042 cells

**Figure 5:**
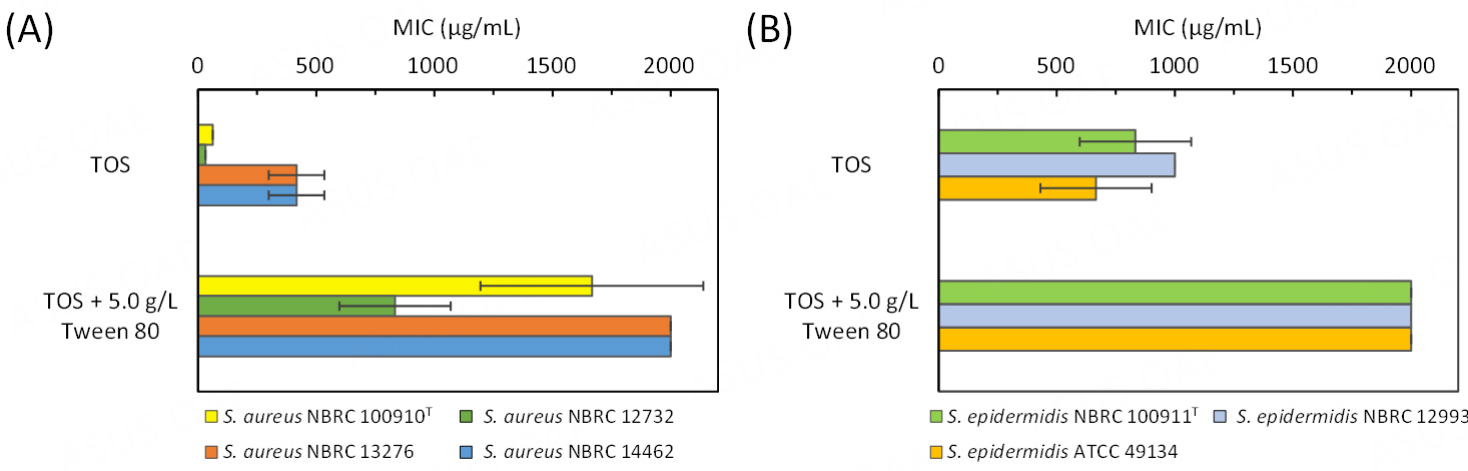
Antibacterial activity of TFA extracted from the JCM 7042 strain against *S. aureus* (A) and *S. epidermidis* (B) strains. The TFA were used as the form of acid-hydrolyzed FFAs from total lipids. FFA: Free fatty acid; TFA: total fatty acids.

Finally, an antibacterial assay was performed using acid-hydrolyzed TFA preparations obtained from the cells of the JCM 7042 strain [Figure 5]. To understand how the presence of Tween 80 in the medium affects lipid metabolism and antibacterial activity, the strain was grown either in TOS medium or in TOS medium supplemented with 5 g/L of the surfactant, and used for TFA preparation.

With respect to the TFA prepared from cells grown in TOS, high antibacterial activity was observed against *S. aureus* NBRC 100910^T^ and NBRC 12732 with MICs of 62.5 µg/mL and 31.3 µg/mL, respectively, while the activity was not very high against *S. aureus* NBRC 13276 and NBRC 14462 with MICs of about 400 µg/mL for both strains. The MIC values towards *S. epidermidis* were in a range between 660 and 1000 µg/mL. The TFA prepared from cells grown in TOS + Tween 80 did not show marked antibacterial activity for *S. aureus* NBRC 100910^T^, NBRC 13276, and NBRC 14462 (MICs >1660 µg/mL), while it showed weak activity towards NBRC 12732 with a MIC of about 830 µg/mL. No activity was observed against *S. epidermidis* (MICs of > 2000 µg/mL).

Fatty acid composition analysis revealed that the addition of Tween 80 to the medium not only decreased the total amount of TFA by 2-fold but also reduced the ratio of C12:0, C14:0, 7-*cis*-C16:1, and 9-*cis*-C16:1 and increased C16:0 and 9-*cis*-C18:1 in TFA [Table 2]. The low abundance of each C16:1, especially 7-*cis*-C16:1, could be responsible for the low antibacterial activity of the TFA prepared from the cells grown in the presence of Tween 80.

**Table 2:** Fatty acid compositions of TFA samples obtained from the JCM 7042 strain cultured in TOS or TOS + 5.0 g/L Tween 80

	**TFA** **(mg/L)**	**Fatty acid ratio (%) in TFA**							
		**C12:0**	**C14:0**	**C16:0**	**7-*cis*-C16:1**	**9-*cis*-C16:1**	**C18:0**	**9-*cis*-C18:1**	**Others^*a*^**
TOS	23.1	4.5	21.9	26.7	6.1	0.3	1.5	18.7	20.3
TOS + 5.0 g/L Tween 80	12.0	0.2	0.7	39.2	0.1	0	2.3	39.5	18.1

a Others include some fatty acids with small ratios in the TFA, such as C10:0, C17:0, *trans*-C18:1, cycloC19, etc; TFA: total fatty acids.

## DISCUSSION

In this study, we revealed that *Bifidobacterium* sp. JCM 7042 produces 7-*cis*-C16:1. The fatty acid was not detected or is produced under the detection limit for other species, such as *B. bifidum*, *B. catenulatum*, *B. indicum*, *B. longum*, and *B. pseudocatenulatum*. Moreover, C16:1-producing ability varied even within the same species, suggesting that there are differences in genetic and/or enzymatic contexts involved in fatty acid biosynthesis within bifidobacterial species and strains. Furthermore, when the JCM 7042 strain was cultured in TOS medium containing Tween 80, the amount of 7-*cis*-C16:1 and its ratio in TFA considerably decreased [Figure 4]. It suggests that 9-*cis*-octadecaenoic acid (oleic acid), which is the intramolecular structure of Tween 80, might be taken into the cells and suppress the biosynthesis pathway of 7-*cis*-C16:1 as feedback inhibition^[39]^. However, studies on fatty acid biosynthesis in *Bifidobacterium* species are limited, and the mechanisms underlying the synthesis and regulation of fatty acids remain elusive. Comparative genomic and transcriptomic analyses, together with detailed fatty acid composition analysis under the varied cultivation conditions, are necessary to obtain a better understanding of fatty acid metabolism in bifidobacteria and the related bacteria belonging to the family *Bifidobacteriaceae*.

Some microorganisms, such as *Aeromonas hydrophila* (*A. hydrophila*), were previously shown to produce 7-*cis*-C16:1 *via *β-oxidation^[40]^. However, because *A. hydrophila* is classified as biosafety level 2 bacterium, its use for cosmetics and food production needs extensive caution. On the other hand, *Bifidobacterium* strains have extensive food experience and are generally regarded as safe. *Lactobacillus* species were previously reported to produce only 9-*cis*-C16:1^[13,41,42]^, and we also examined more than 10 strains of *Lactobacillus *strains. However, they did not produce 7-*cis*-C16:1 under the conditions tested (data not shown). Examining the prevalence of 7-*cis*-C16:1 in various intestinal microorganisms is also necessary to predict the link between the health benefits of the fatty acid and gut microbiota.

Antibacterial assays using authentic C16:1 standards and TFA prepared from the JCM 7042 strain revealed that, in addition to their high antibacterial activity against *S*. *aureus*, the activity against *S*. *epidermidis* can vary depending on the position of the double bond [Figure 3] and can also be probably affected by the presence of other fatty acids as discussed below [Figure 5]. The position of double bond seemed to affect the antibacterial activity against *S. epidermidis*, in which activity became high when the double bond was closer to the methyl end of the fatty acid. Taken together, C16:1 may be a key determinant of the antibacterial selectivity of *S. aureus*.

The mechanisms of antibacterial activity of C16:1 against *S. aureus* have been reported previously. Treatment with 9-*cis*-C16:1 caused rapid membrane depolarization, the disruption of all major branches of macromolecular synthesis, and the release of solutes and low molecular weight proteins into the medium in* S. aureus *cells^[43,44]^. In addition, *S. aureus* exhibits membrane lipid plasticity, which is of potential relevance to the response and resistance to various antimicrobial agents. It has been reported that different proportions of branched-chain and straight-chain fatty acids have been observed between *S. aureus* and coagulase-negative staphylococci^[45]^. The differences in their membrane structure may be related to the selective antibacterial activity of C16:1 against *S. aureus* and *S. epidermidis*.

The antibacterial activity of TFA obtained from the JCM 7042 cells grown in the TOS medium against *S. aureus* NBRC 13276 and NBRC 14462 was weaker than that against *S. aureus* NBRC 100910^T^ and NBRC 12732 [Figure 5]. *S. aureus *NBRC 14462 also showed higher tolerance than the other three strains to the authentic 7-*cis*-C16:1 [Figure 3]. It is reported that the adulteration of fatty acid samples with oleic acid (C18:1) inhibited the antibacterial activity against *S. aureus* NBRC 13276 and NBRC 14462^[46]^. The TFA sample obtained from the JCM 7042 strain cultured in the TOS medium contained 18.7% oleic acid and showed weaker activity against *S. aureus* NBRC 13276 and NBRC 14462 than *S. aureus* NBRC 100910^T^ and NBRC 12732. Nonetheless, the TFA sample obtained from the JCM 7042 strain cultured in the TOS medium showed stronger activity than the TFA sample obtained from the JCM 7042 strain cultured in a TOS medium containing Tween 80. Therefore, the presence of 7-*cis*-C16:1 is indeed pivotal for the TFA preparation to exert its antibacterial activity against *S. aureus*. The presence of C14:0 could also be effective in increasing the activity of 7-*cis*-C16:1 because it has been shown that C14:0 possesses weak antibacterial activity and that, when mixed with C16:1, it shows synergistic antibacterial activity against *S. aureus*^[47]^. All of these results warrant further research on TFA prepared from the JCM 7042 strain as a material for practical use, which includes the establishment of culture conditions for increasing 7-*cis*-C16:1 and C14:0 while decreasing C18:1. It should be noted that the TFA prepared from the JCM 7042 strain have moderate antibacterial activity against *S*. *epidermidis *[Figure 5]. *S*. *epidermidis *is a common commensal microorganism on skin and prevents the *S. aureus* growth, but the overabundance of *S*. *epidermidis* among patients with atopic dermatitis can cause skin damage by extracellular proteases, similar to *S*. *aureus*^[48]^. A tuned balance in the microbiota is thus considered to be important.

We used 4 strains of *S. aureus* and 3 strains of *S. epidermidis* in this study and found that, even in the small number of strains examined, the antibacterial activity of 7-*cis*-C16:1 and the extracted TFA against the staphylococci vary. Future research should include clinical isolates from patients with atopic dermatitis and multidrug-resistant (methicillin-resistant) *S. aureus* (MRSA) associated with bacteremia. Nonetheless, it is noteworthy that C16:1 isomers, especially 6-*cis*-C16:1 and 7-*cis*-C16:1, possess considerable high selectivity towards *S. aureus*. It is a recent clinical concept to suppress only harmful microorganisms associated with diseases but to coexist with beneficial microorganisms that contribute to maintaining health^[49]^. We hope that *Bifidobacterium* and its fatty acids described herein will provide a basis for developing a novel therapeutic approach to treating and preventing *S. aureus*-associated disorders and for opening up a way to its use in cosmetics and functional foods.

## DECLARATIONS

### Copyright

© The Author(s) 2023.
